# Diagnostic impact and reproducibility of p53 immunohistochemistry in Barrett's oesophagus: results of the Dutch Esophageal Pathology Panel (DEPP)

**DOI:** 10.1111/his.70193

**Published:** 2026-06-19

**Authors:** Ylva A. Weeda, Sarah M. Issa, Hans Halfwerk, Michel Botros, Onno J. de Boer, Lodewijk A. Brosens, Michail Doukas, Gursah Kats‐Ugurlu, Fiebo ten Kate, Jaap van der Laan, Ineke van Lijnschoten, Ariadne H. A. G. Ooms, Lindsey Oudijk, Rachel S. van der Post, Mihaela G. Raicu, Bert Timmer, Myrtle J. van der Wel, Sybren L. Meijer

**Affiliations:** ^1^ Department of Pathology Amsterdam University Medical Center at the University of Amsterdam Amsterdam the Netherlands; ^2^ Cancer Center Amsterdam, Biomarkers Amsterdam the Netherlands; ^3^ Department of Biomedical Engineering and Physics Amsterdam University Medical Center at the University of Amsterdam Amsterdam the Netherlands; ^4^ Department of Pathology University Medical Center Utrecht, Utrecht University Utrecht the Netherlands; ^5^ Department of Pathology Erasmus University Medical Center Rotterdam the Netherlands; ^6^ Department of Pathology and Medical Biology University Medical Center Groningen Groningen the Netherlands; ^7^ Department of Pathology Isala Klinieken Zwolle the Netherlands; ^8^ Department of Pathology HAGA Hospital The Hague the Netherlands; ^9^ Catharina Hospital, PAMM Eindhoven the Netherlands; ^10^ Pathan BV Rotterdam the Netherlands; ^11^ Department of Pathology Maasstad Hospital Rotterdam the Netherlands; ^12^ Department of Pathology Radboud University Medical Center Nijmegen the Netherlands; ^13^ Department of Pathology St. Antonius Hospital Nieuwegein the Netherlands

**Keywords:** Barrett's oesophagus, gastrointestinal pathology, p53‐immunohistochemistry, tissue diagnosis

## Abstract

**Introduction:**

Evidence supporting the use of p53 immunohistochemistry (p53‐IHC) in Barrett's oesophagus (BE) is largely based on selected series, limiting generalizability. This study evaluates p53‐IHC in a nationwide, real‐world BE‐cohort within a universal healthcare system, assessing concordance with referring hospitals, interobserver agreement (IOA) and its impact on routine diagnostic decisions.

**Methods:**

Revision cases submitted to the Dutch Esophageal Pathology Panel (DEPP) were assessed by trained and experienced pathologists for haematoxylin and eosin (H&E)‐based classification (Vienna classification), p53‐IHC patterns (wild‐type, overexpression, double clone, null mutation and equivocal) and combined H&E/p53‐IHC review. Concordance between referring hospitals and the expert panel (Cohen's kappa) and IOA among panel pathologists (Fleiss' kappa) were assessed, along with the impact of p53‐IHC on diagnostic reclassification.

**Results:**

A total of 1,146 consecutive patient consultation cases (1,704 biopsy levels) were included, with a median of 12 DEPP pathologist assessments per biopsy. Overall concordance of p53 assessment between referring hospitals and the expert panel was moderate‐to‐substantial (*κ* = 0.61, 95% CI 0.58–0.64), with 24.2% of p53‐IHC assessments reclassified. IOA among 13 pathologists assessing 316 endoscopic biopsies was substantial (*κ* = 0.65, 95% CI 0.61–0.69). Ancillary p53‐IHC influenced pathologists' decisions across biopsy levels. For non‐dysplastic (NDBE) biopsies, aberrant p53‐IHC prompted reclassification to indefinite for dysplasia (IND, 9.2%) or low‐grade dysplasia (LGD, 4.8%). For IND biopsies, p53‐IHC shifted diagnoses toward NDBE (17.0%) or LGD (37.1%).

**Conclusion/discussion:**

p53‐IHC interpretation is reliable, reproducible and clinically meaningful in BE diagnostics when standardized criteria are applied. These findings support broader use of p53‐IHC in routine practice and incorporation of assessment criteria into guidelines.

## Graphical abstract

Structured p53‐IHC in Barrett's oesophagus supports diagnostic decision‐making. In 1,146 cases, agreement between referring hospitals and the expert panel was moderate‐to‐substantial (*κ* = 0.61), with 24.2% of p53‐based classifications revised. Interobserver agreement among expert pathologists was substantial (*κ* = 0.65). P53‐IHC prompted re‐evaluation of morphology‐based NDBE and supported triage of morphology‐based IND cases in individual pathologist decision‐making.

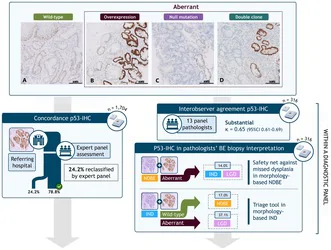

**Abbreviations**
BE: Barrett's oesophagus
CIs: confidence intervals
DC: double clone
DEPP: Dutch Esophageal Pathology Panel
EQ: equivocal
H&E: haematoxylin and eosin
HGD: high‐grade dysplasia
IND: indefinite for dysplasia
IOA: interobserver agreement
LGD: low‐grade dysplasia
NDBE: non‐dysplastic BE
NM: null mutation
OE: overexpression
OGD: oesophagogastroduodenoscopy
p53‐IHC: p53 immunohistochemistry
WT: wild‐type

## Introduction

Histopathological assessment of Barrett's oesophagus (BE) biopsies is challenging and subject to interobserver variability directly affecting patient management and outcome. Therefore, national and international guidelines recommend expert histopathology review for dysplastic cases.^1^, ^2^ In the Netherlands, this review is facilitated by the Dutch Esophageal Pathology Panel (DEPP): an expert panel for reviewing ambiguous BE cases. The panel comprises experienced pathologists from nine leading expert centres, who have all completed rigorous training programs prior to joining.^3^, ^4^

In addition to expert review, the immunohistochemical staining for tumour suppressor protein p53 (p53‐IHC) has shown value to improve diagnostic reproducibility in BE.^5^, ^6^, ^7^, ^8^, ^9^, ^10^ Despite evidence that p53‐IHC can enhance interobserver agreement, its widespread clinical implementation remains limited,^8^ mainly due to the lack of standardized criteria for aberrant staining and concerns regarding interobserver variability.^5^, ^11^, ^12^, ^13^ Previous studies assessing p53‐IHC interobserver variability have used heterogeneous designs, limiting comparability and generalizability.^5^, ^12^, ^13^

In the Netherlands, p53‐IHC is used with a low threshold and is commonly reported in BE diagnostics.^14^ Evaluation of 1,146 consecutive cases reviewed within the DEPP cohort therefore provides a unique opportunity to assess the added value of p53‐IHC in daily practice, in a standardized, multi‐expert setting. First, we compare p53‐IHC results reported by referring hospitals with the standardized assessment performed by the DEPP. Next, we evaluate the interobserver agreement of p53‐IHC among DEPP pathologists. Finally, we examine the impact of p53‐IHC assessment on diagnostic decision‐making within this panel and overall patient management.

## Methods

### Patients and Samples

This study included a consecutive review of 1,146 patients who underwent oesophagogastroduodenoscopy (OGD) for BE screening or surveillance between January 2015 and February 2025. A total of 39 referring, community‐based hospitals submitted endoscopic biopsy slides for central review at the Amsterdam UMC, primarily for cases with diagnostic uncertainty such as suspected indefinite for dysplasia (IND) or low‐grade dysplasia (LGD). All referring centres operate within accredited pathology departments and participate in external quality assurance programs. Slides were digitized at 40× magnification (0.25 microns per pixel) using a Philips Ultra‐Fast Scanner (Philips Digital Pathology Solutions, Best, The Netherlands) slide, assessed for quality according to standard digital workflow protocols, and uploaded to a web‐based whole slide image viewer (Tutor, Cirdan, Lisburn, N. Ireland). Endoscopic biopsies without reported or performed p53‐IHC at the referring hospital were excluded from analysis.

### Panel Assessment and Histological Assessment of Revision Cases

The DEPP currently consists of 13 participating pathologists with 5–30 years of experience. Over time, panel membership has evolved, with pathologists leaving and new members joining. Despite these changes, a minimum of five members was maintained throughout, ensuring consistent evaluation capacity. Consequently, not all cases were assessed by the same individuals. In total, assessments from up to 18 pathologists are included in the dataset. Before joining the panel, pathologists completed mandatory quantitative training programs with predefined benchmark criteria to ensure reliable and standardized histopathological assessment.^3^, ^4^

#### H&E morphology

Each endoscopic biopsy level was classified according to the modified Vienna criteria for gastrointestinal neoplasms into four categories: non‐dysplastic BE (NDBE), IND, LGD and high‐grade dysplasia (HGD). Samples suspicious for invasive carcinoma were grouped within the HGD category.^15^ Panel pathologists provided a diagnosis based on the H&E slides alone and again after review of H&E slides together with p53‐IHC.

#### p53‐IHC classification

**Figure 1:**
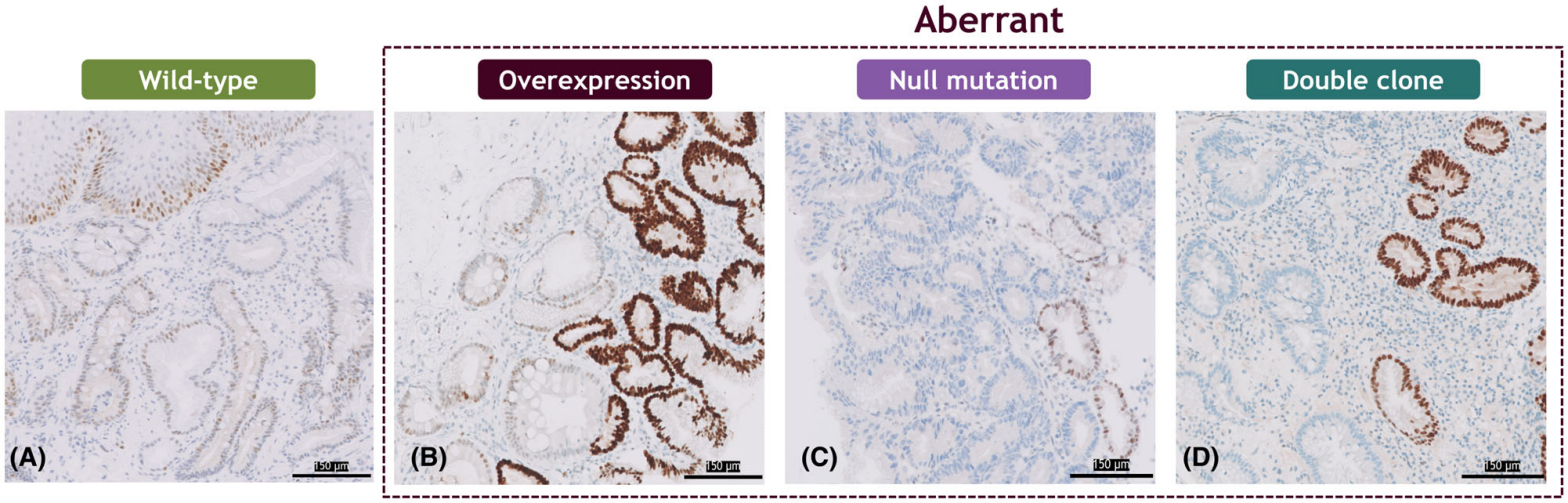
Representative examples of standardized p53‐IHC staining patterns (wild‐type, overexpression, null mutation and double clone). Scale bars are equal to 150 microns. [Colour figure can be viewed at wileyonlinelibrary.com]

p53‐IHC staining was classified into four patterns: wild‐type (WT), overexpression (OE), null mutation (NM) and double clone (DC). WT was characterized by weak to moderate, heterogeneous nuclear staining in a mottled pattern. OE was defined as strong nuclear staining involving at least one entire crypt (or the equivalent, depending on the plane of section), adapted from Tomaszewski *et al*.^16^ Staining intensity was required to exceed that of the basal layer of the squamous epithelium (when present) or the adjacent epithelium.^16^ NM was defined as a complete absence of nuclear staining.^17^ DC referred to the coexistence of NM and OE patterns within the same biopsy. For part of the analysis, OE, NM and DC patterns were combined and considered ‘aberrant’^18^, ^19^ (Figure 1).

##### Referring hospitals

p53‐IHC results from referring hospitals were extracted from pathology sign‐out reports. Patterns reported as unclear or ambiguous in these reports were classified as ‘equivocal’ (EQ).

##### Panel

p53‐IHC assessment within the panel was standardized. Individual panel pathologist assessments were recorded, and final panel consensus required 100% agreement among three pathologists, ≥75% agreement among four pathologists and ≥70% when more than four pathologists reviewed the case. Slides not meeting these thresholds were reviewed by four additional pathologists and unresolved cases were deemed EQ.

### Statistical Analysis

Concordance between the referring hospital and the expert panel p53‐IHC classification was assessed using descriptive statistics (agreement proportions, rates of alteration) and Cohen's kappa. Ninety‐five per cent confidence intervals (CIs) were estimated using bootstrapping with 10,000 iterations.

Interobserver agreement for p53‐IHC among experienced pathologists was evaluated in the largest subset of cases reviewed by the maximum number of overlapping pathologists, resulting in a dataset without missing values. Fleiss' kappa was calculated for the five p53 categories (WT, OE, DC, NM and EQ), with CIs derived using the same bootstrapping approach. Diagnostic shifts induced by p53‐IHC were quantified by extracting each pathologist's H&E, p53‐IHC, and combined H&E/p53‐IHC assessments. Unique diagnostic combinations were pooled across pathologists and aggregated within the complete‐case subset to determine how often H&E interpretations changed after incorporation of p53‐IHC. Combinations occurring in <0.5% of samples were excluded. All analyses have been performed using SPSS V.28 (SPSS Inc, Chicago, Illinois, USA) and Python (version 3.10; Python Software Foundation, Wilmington, DE, USA).

## Results

**Figure 2:**
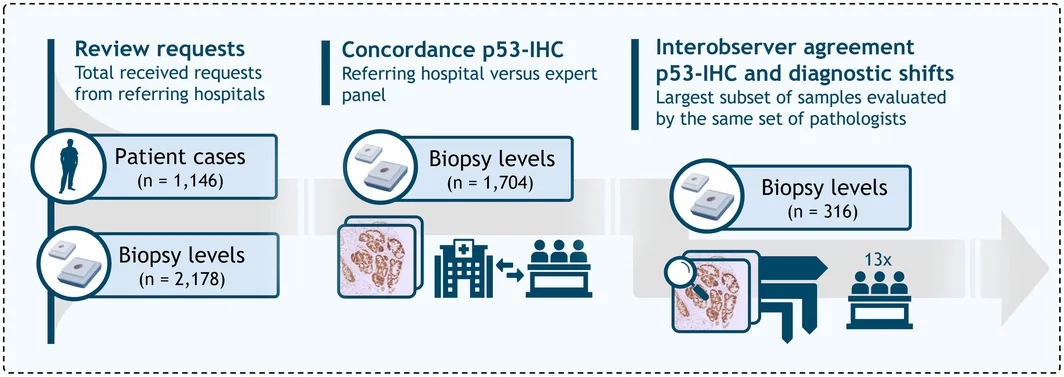
Flow of review requests and subsets used for analysis. 1,146 revision requests (2,178 endoscopic biopsy levels) were received from 39 hospitals. Concordance analysis included 1,704 biopsy levels with available p53‐IHC and corresponding classifications reported by both the referring hospital and the expert panel. Interobserver agreement and diagnostic shifts were assessed in the largest fully complete subset of cases with identical raters, comprising 316 endoscopic biopsy levels reviewed by 13 pathologists. [Colour figure can be viewed at wileyonlinelibrary.com]

Between January 2015 and February 2025, the DEPP received 1,146 consecutive review requests from 39 referring hospitals. Each patient case included a median of two endoscopic biopsy levels (range: 1.0–8.0), resulting in a total of 2,178 evaluated endoscopic biopsy levels. After excluding cases without ancillary p53‐IHC (*n* = 403) or with missing p53 results from the sign‐out report (*n* = 71), 1,704 biopsy levels were eligible for concordance analysis between referring hospitals and the DEPP. For interobserver agreement and diagnostic shift analyses within the DEPP, the largest subset of samples evaluated by the same set of pathologists (316 biopsy levels scored by 13 pathologists) was selected to avoid missing data (Figure 2).

### Concordance p53‐IHC Referring Hospitals Versus DEPP Assessment

**Figure 3:**
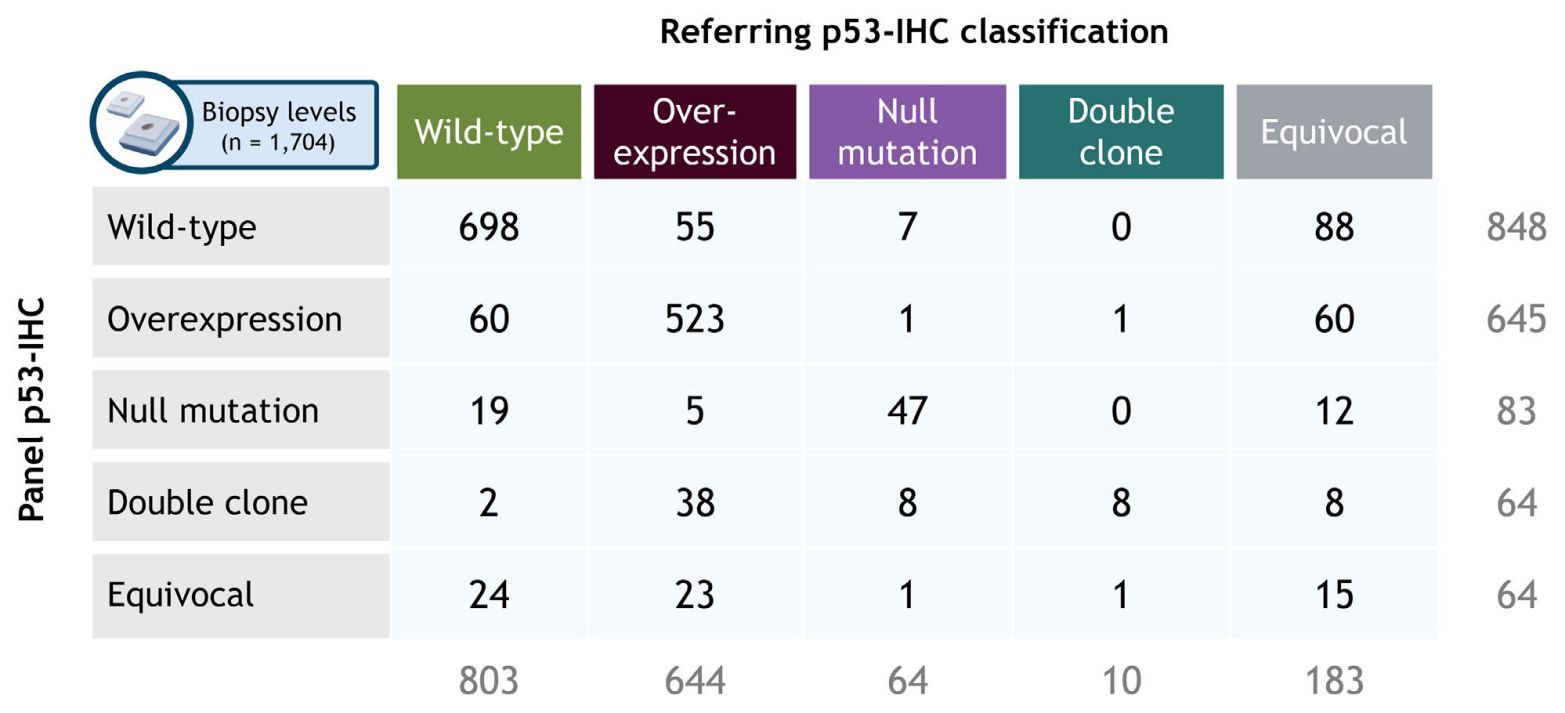
Contingency table comparing the referring hospital's p53‐IHC classification with the consensus panel's p53‐IHC assessment for all endoscopic biopsy levels with available H&E and p53‐IHC staining (*n* = 1,704). Values along the diagonal indicate agreement between the referring hospital and the panel. Overall agreement was 75.8% (1,291/1,704). [Colour figure can be viewed at wileyonlinelibrary.com]

**Figure 4:**
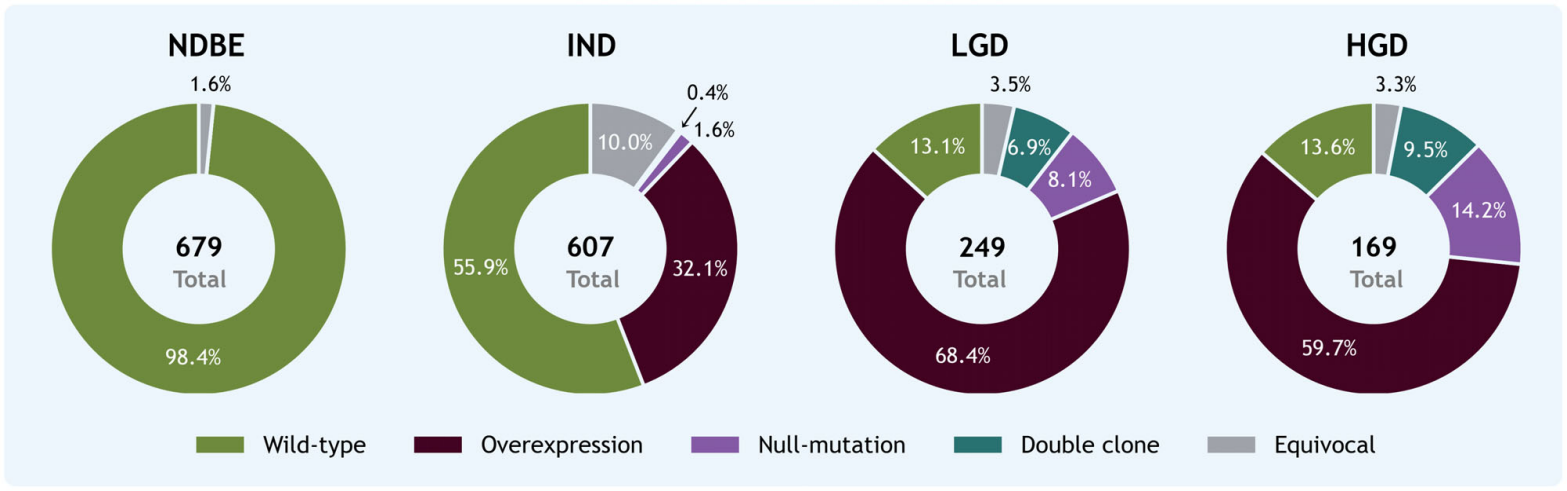
Donut plot showing the distribution of p53 patterns for each Vienna classification category. Both the p53 patterns and Vienna categories were defined based on expert panel consensus. HGD, high‐grade dysplasia; IND, indefinite for dysplasia; LGD, low‐grade dysplasia; NDBE, non‐dysplastic Barrett's oesophagus.

The referring hospital classified the endoscopic biopsy levels (*n* = 1,704) as 803 WT (47.1%), 644 OE (37.8%), 64 NM (3.8%), 10 DC (0.6%) and 183 EQ (10.7%) samples. Each biopsy was evaluated by a median of 12 DEPP pathologists (range: 3–18), with each pathologist assessing a median of 843 (range: 113–1,699) samples. Review by DEPP resulted in reclassification of p53 IHC in 24.2% of all samples. Overall concordance between referring hospitals and DEPP was moderate‐to‐substantial, with a Cohen's kappa of 0.61 (95% CI: 0.58–0.64). The lowest discordance rate occurred in the WT (13.1%) and OE (18.8%) categories, although misclassifications between these two patterns still occurred. Namely, 8.5% (55/644) of OE samples were changed to WT by the panel, and 7.5% (60/803) of WT to OE. For the NM and DC categories, the discordance rates were higher, namely 26.6% and 20.0%, respectively. The NM pattern was frequently overlooked, as 36 samples initially classified as WT (19/83), OE (5/83) or EQ (12/83) were reclassified as the NM pattern. Most EQ samples (91.8% of 183 cases) were recategorized by the panel, predominantly as WT (48.1%) or OE (32.8%). All frequencies described above are visualized in the contingency table in Figure 3. The distribution of p53 expression patterns across the different Vienna‐classified morphological diagnoses, as determined by the expert panel, is shown in Figure 4.

### Interpretation of Equivocal p53‐IHC Patterns

EQ cases represent biopsies in which aberrant p53 staining could not be determined with sufficient diagnostic confidence. Within the DEPP panel, 3.8% of biopsies (64/1,704) were classified as EQ, meaning that no consensus could be reached despite standardized multi‐expert review. Diagnostic uncertainty typically resulted from combined staining‐related technical factors (e.g., tissue folds, background staining) and morphological ambiguity, complicating interpretation of borderline patterns such as limited crypt involvement or heterogeneous mosaic expression (Figure S1A,B).

### Interobserver Agreement p53‐IHC Within a Diagnostic Panel

**Figure 5:**
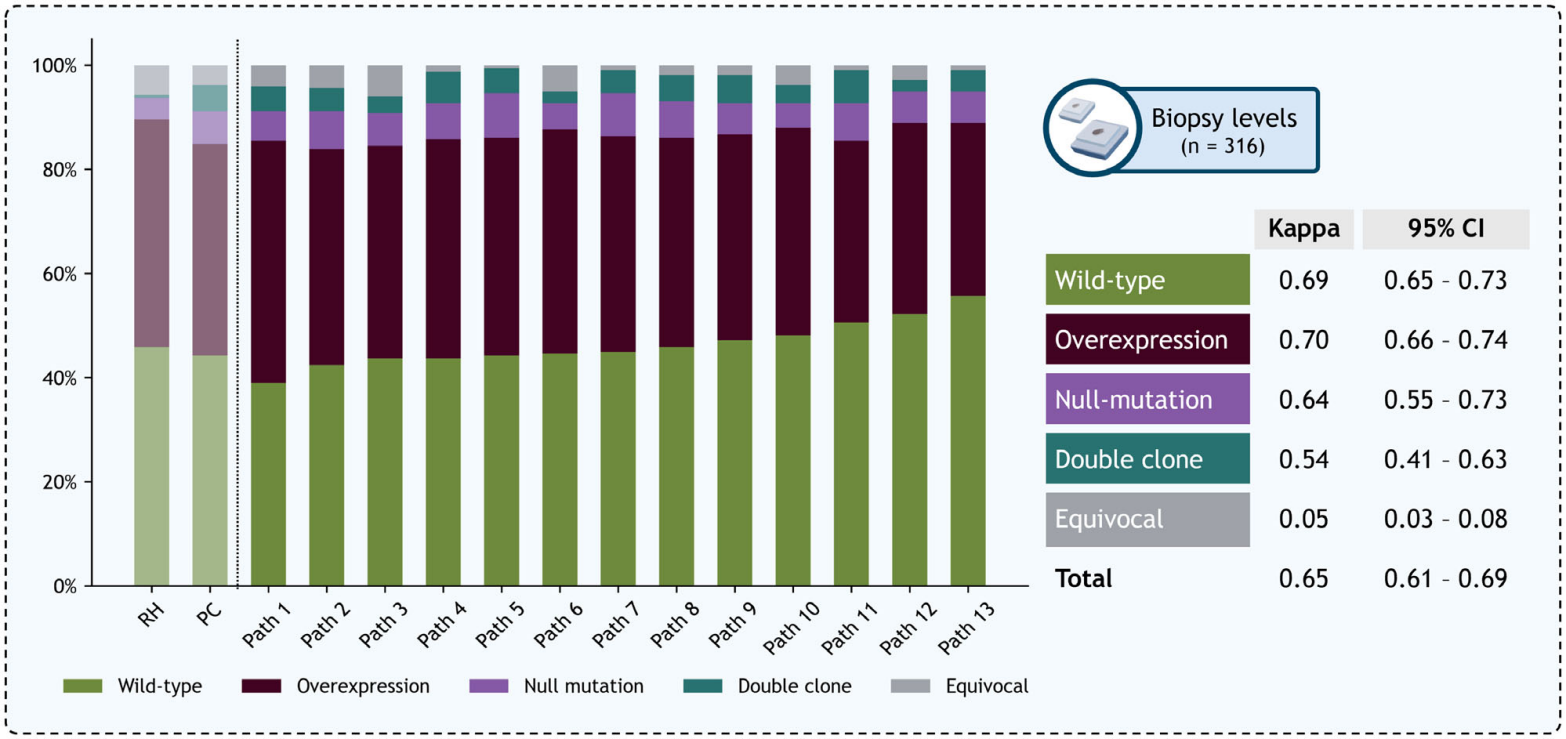
Bar plot showing p53‐IHC agreement among 13 panel pathologists for 316 endoscopic biopsy levels. The proportions of p53 classifications from the referring hospitals and the panel consensus are also displayed in a transparent format for comparison. Interobserver agreement for all p53‐staining patterns was quantified using Fleiss' kappa and is presented in the table adjacent to the bar plot. PC, panel consensus; RH, referring hospital.

From the total cohort of 1,704 endoscopic biopsy levels, a subset of 316 biopsy levels evaluated by the same group of 13 pathologists was identified. The subset enabled the quantification of interobserver agreement for p53‐IHC assessment (Figure 5). For the five individual p53 categories (WT, OE, DC, NM, and EQ), the overall Fleiss' kappa was 0.65 (95% CI 0.61–0.69). Agreement was substantial for the WT (*κ* = 0.69, 95% CI 0.65–0.73) and OE (*κ* = 0.70, 95% CI 0.66–0.74) categories, moderate‐to‐substantial for NM (*κ* = 0.64, 95% CI 0.55–0.73), and moderate in DC (*κ* = 0.54, 95% CI 0.41–0.63). The lowest interobserver agreement was computed for the EQ category (*κ* = 0.05, 95% CI; 0.03–0.08). Excluding EQ cases increased kappa to 0.74 (95% CI: 0.70–0.78), demonstrating substantially higher agreement for the remaining cases (*n* = 243).

### Impact of p53‐ IHC on Diagnostic Decisions by Individual Panel Pathologists

The largest subset of cases evaluated by the maximum number of overlapping pathologists contained 4,108 ratings (316 biopsy levels × 13 pathologists). All resulting H&E, p53‐IHC, and diagnostic combinations were aggregated, with p53‐IHC categories collapsed into WT, aberrant (OE, NM and DC), and EQ to improve interpretability. After excluding rare combinations (occuring in <0.5% of samples), 14 diagnostic combinations were observed across all pathologists and biopsy levels. All combinations are provided in Data S1.

**Figure 6:**
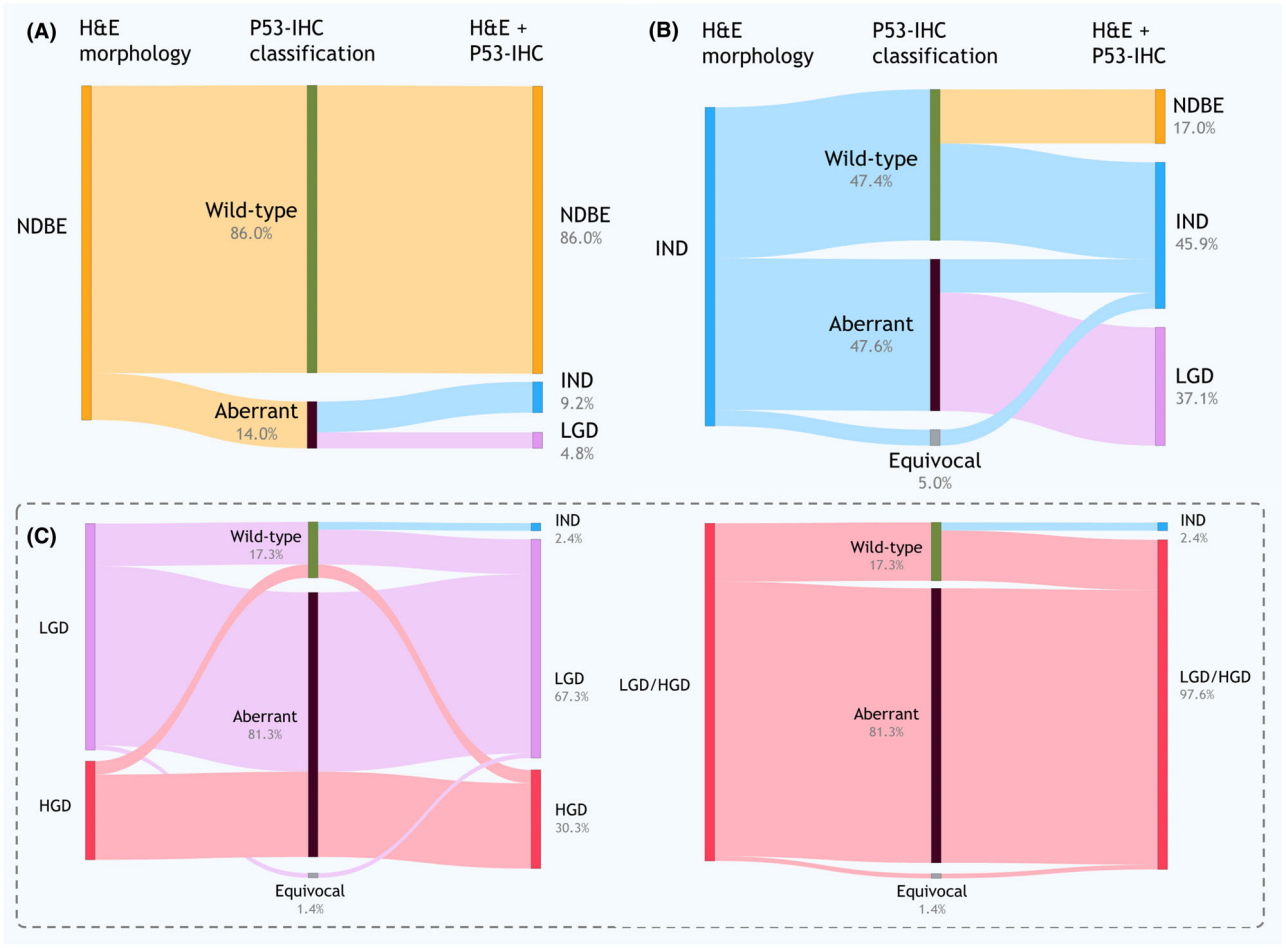
Sankey diagrams were used to visualize diagnostic shifts in an aggregation of 4,108 ratings among 13 DEPP pathologists, showing how ancillary p53‐IHC testing influenced individual pathologists' H&E‐based assessments, illustrating the transition to the combined H&E/p53‐IHC assessment. (**A**) Non‐dysplastic Barrett's Oesophagus (NDBE), (**B**) indefinite for dysplasia (IND), and (**C**) low‐grade dysplasia (LGD) and high‐grade dysplasia (HGD), shown separately and combined to demonstrate minimal impact of p53‐IHC within the LGD/HGD spectrum. [Colour figure can be viewed at wileyonlinelibrary.com]

Most H&E‐based NDBE diagnoses by individual DEPP pathologists remained unchanged following p53‐IHC assessment, as WT p53 staining pattern did not prompt diagnostic revision (86.0%). In the remaining 14.0%, combined assessment with focal ancillary aberrant p53‐IHC staining prompted individual pathologists to reclassify from NDBE to LGD (4.8%) or IND (9.2%) (Figure 6A). Illustrative examples show biopsies initially classified as NDBE by individiual pathologists, where subsequent p53‐IHC revealed subtle focal aberrant staining prompting reclassification (Figure S2).

Ancillary p53‐IHC had the greatest impact on samples initially diagnosed on H&E as IND by individual panel pathologists, with alterations occurring in 54.1%. Specifically, 17.0% of IND diagnosed on H&E were reclassified as NDBE when combined with a WT p53 staining, and 37.1% as LGD in the presence of aberrant p53 staining. In the remaining 45.9% of IND cases, the diagnosis remained unchanged, irrespective of p53‐IHC staining patterns (Figure 6B).

In LGD and HGD samples, only a minority of samples with WT p53‐IHC were reclassified as IND (2.4%, Figure 6C).

## Discussion

This study highlights the added value of p53‐IHC in BE management. Concordance between referring hospitals and the expert panel was moderate‐to‐substantial, although some variation persisted. Interobserver agreement among experienced pathologists was substantial, and p53‐IHC supported clinically relevant diagnostic reclassification by influencing individual pathologists' assessments. p53‐IHC has the potential to reduce underdiagnosis of dysplasia and demonstrates added value in both general GE‐pathology practice and expert assessment.

To our knowledge, this is the first study to directly compare p53‐IHC interpretation in daily routine pathology practice with expert panel assessment. The overall concordance with the DEPP was moderate‐to‐substantial (*κ* = 0.61, 95% CI: 0.58–0.64). Nearly one quarter of all p53‐IHC biopsy assessments (24.2%, 413/1,704) were reclassified by the panel. A substantial proportion of discordance was driven by recategorization of referring hospital EQ p53 interpretations (9.9%, 168/1,704) as a definitive WT or aberrant staining pattern. In general GE pathology practice, misclassification between WT and OE patterns occured in 6.7% of samples (115/1,704), but did not account for the largest proportion of reclassifications. In addition, the NM pattern was frequently missed (36/83) by referring hospitals. These findings indicate that while p53‐IHC interpretation can be reliable, it can also be further improved through standardization and training.

The absolute lower limit of aberrantly stained nuclei required to classify p53‐IHC as aberrant is difficult to define. While Tomaszewski *et al*.^16^ recommended considering either one crypt or five consecutive positive nuclei as sufficient to define aberrant p53‐IHC, we used a simplified definition requiring involvement of at least one crypt, regardless of the number of positive nuclei. Using this criterion, interobserver agreement among the 13 DEPP pathologists in the subset analysis was substantial across five diagnostic categories. Previously reported kappa values ranged from 0.60 to 0.83,^5^, ^12^, ^13^ although direct comparison is challenging due to differences in statistical methods, p53‐IHC classification criteria, number of pathologists involved, and case numbers evaluated. Our study extends these findings by including more pathologists, multiple centres, a large consultation‐based case load, and an additional fifth ‘equivocal’ category.

This EQ category was presented as an additional sign‐out option inspired by general pathology practice, as real‐world experience shows that some p53‐IHC patterns are hard to definitively classify. Biopsies initially labelled EQ by referring hospitals were mostly resolved after panel review, with only 3.8% remaining EQ. EQ cases fall within a biological and interpretative grey zone, in which focal nuclear positivity may approach but not fully meet the criteria of complete crypt involvement. This emphasizes that p53‐IHC interpretation inherently involves a degree of subjectivity and that even with structured expert review a small proportion of cases will remain ambiguous. Although molecular analyses could potentially clarify the TP53 status, they are not routinely performed within the DEPP due to cost constraints. Nonetheless, these observations underscore the value of panel assessment, and we recommend that EQ p53 cases be submitted for review.

Inaccurate initial p53‐IHC assessment at the referring hospital contributed to a limited number of clinically relevant misclassifications, with implications for surveillance intervals or therapeutic decision‐making. Of the biopsies in which p53‐IHC was revised by the DEPP, only 6.7% (115/1,704) resulted in diagnostic shifts with clinically relevant patient‐level consequences. The two main drivers of these diagnostic shifts were recategorization of EQ p53‐IHC (*n* = 50) and misinterpretation of WT and aberrant p53‐IHC (*n* = 58). Misclassification between WT and OE patterns likely arises from interpretative challenges, with increased background or mosaic staining patterns leading to overcalling as OE, and focal or gland‐restricted staining causing undercalling of OE as WT. Clinically relevant reclassification followed a consistent pattern. Reassignment from EQ or WT to aberrant p53‐IHC was associated with reclassification from NDBE to IND or LGD, and from IND to HGD, prompting escalation of clinical management. Conversely, reclassification from EQ or aberrant p53 to WT predominantly resulted in reclassification from IND or LGD to NDBE, leading to de‐escalation of surveillance intensity. Detailed results on diagnostic shifts are provided in the Data S1. More accurate upfront p53‐IHC interpretation could enable appropriate risk stratification, prevent unnecessary patient anxiety, and reduce healthcare costs. Targeted educational efforts in referring hospitals can further improve awareness of staining patterns, reduce misclassifications, and enhance the added value of p53‐IHC in patient management.

Based on individual DEPP pathologist assessments in this study, p53‐IHC interpretation has a direct impact on diagnostic decision‐making within this panel. In biopsies with NDBE morphology on H&E, ancillary aberrant p53‐IHC staining prompted individual pathologists to re‐evaluate the H&E slides. This led to reclassification as LGD (4.8%) or IND (9.2%), when subtle morphological dysplastic features were retrospectively identified on H&E. In this setting, p53‐IHC enhances the detection of focal and subtle dysplasia that would otherwise be overlooked or flags biopsies with aberrant p53 signatures as IND. In biopsies signed out as IND on H&E, p53‐IHC primarily functioned as a triage tool, where ancillary aberrant staining prompted reclassification as LGD (37.1%), and WT staining as NDBE (17.0%), when concordant with H&E morphology. Of note, IND designates genuinely equivocal histpathology rather than representing an intermediate step in progression toward HGD or EAC. When combined with p53‐IHC, the reclassification of the diagnostic IND subcategory as either NDBE or LGD can further minimize equivocal histopathology assessments and direct unambiguous clinical follow‐up strategies.

A key strength of this study is its scale, as it represents the largest multicentre cohort of consecutive consultation cases with both H&E and p53‐IHC reviewed by a large group of benchmarked pathologists. This provides a robust platform to assess the reproducibility of p53‐IHC. Several limitations should also be acknowledged. Because the cohort consists primarily of diagnostically challenging Dutch consultation cases, the findings may not be generalizable to routine clinical practice worldwide. In addition, subtle variation in p53‐IHC staining protocols across referring hospitals may occur and could have impacted p53‐IHC interpretation. In the Netherlands, laboratories must adhere to quality assurance programs with standardized protocols, including validated antibodies and certified automated staining procedures. As a result, the potential impact of staining variation on our study findings is limited. Future research should include longitudinal progression data in BE patients, with particular attention to subgroups such as LGD and IND cases exhibiting WT versus aberrant p53‐IHC. Integration of molecular analyses could provide greater clarity on equivocal p53 patterns and how to classify these. These strategies would enable more precise risk stratification of BE patients.

Taken together, this study shows that, with standardized interpretation, p53‐IHC is reliable, reproducible, and clinically impactful. We therefore advocate broader use of p53‐IHC in routine BE diagnostics and incorporation of assessment criteria into guidelines.

## Author contributions

Conceptualization: Y.A.W., S.M.I., M.J.W., S.L.M.; data curation: Y.A.W., S.M.I., H.H., O.J.B., M.J.W., S.L.M.; formal analysis: Y.A.W., S.M.I.; funding acquisition: S.L.M.; investigation: Y.A.W., S.M.I.; methodology: Y.A.W., S.M.I., M.J.W., S.L.M.; project administration: Y.A.W., S.M.I., M.J.W., S.L.M.; resources: L.A.B., M.D., G.K.‐U., F.K., J.L., I.L., A.H.A.G.O., L.O., R.S.P., M.C.R., B.T., M.J.W., S.L.M.; software: Y.A.W., S.M.I.; supervision: M.J.W., S.L.M.; validation: Y.A.W., S.M.I., S.L.M.; visualization: Y.A.W., S.M.I., S.L.M.; writing – original draft: Y.A.W., S.M.I., S.L.M.; writing – review and editing: H.H., M.B., L.A.B., O.J.B., L.A.B., M.D., G.K.‐U., F.K., J.L., I.L., A.H.A.G.O., L.O., R.S.P., M.C.R., B.T., M.J.W.

## Funding information

This research was supported by grants from the ‘Koningin Wilhelmina Fonds’ (KWF 150591) and ‘Maag Lever Darm Stichting’ (MLDS WO 21–25). The funders had no role in the design of the study, in the collection, analyses, or interpretation of data, in the writing of the manuscript, or in the decision to publish the results.

## Conflict of interest statement

The author(s) declare that they have no known competing financial or non‐financial interests that could have appeared to influence the work reported in this paper.

## Supporting information


**Data S1.** Supporting information.

**Data S2.** Supporting information.

**Figure S1.** Regions of interest from p53‐stained endoscopic biopsies classified as ‘equivocal’ by the expert panel, reflecting lack of interobserver agreement. (**A**) Isolated single‐crypt involvement (arrow). (**B**) Mosaic p53 expression pattern with abrupt intensity variations.
**Figure S2.** Illustrative examples with morphology resembling NDBE, where subtle focal aberrant p53‐IHC patterns (overexpression (**A**), double clone (**B**), null mutation (**C**)) prompted re‐evaluation by multiple individual pathologists, resulting in diagnostic reclassification.

## Data Availability

The datasets generated and/or analysed during the current study contain sensitive patient information and are not publicly available. Access to pseudonymized data may be granted following a formal data access request and approval in accordance with institutional policies, informed consent, and applicable data protection regulations.
