# Supplementary material for: Diagnostic impact and reproducibility of p53 immunohistochemistry in Barrett's oesophagus: results of the Dutch Esophageal Pathology Panel (DEPP)
Source: Histopathology. 2026 Jun 19;89(4):614–23. doi: 10.1111/his.70193 (PMC13551266; doi:10.1111/his.70193)
Supplement: Supplementary file 1 [file HIS-89-614-s002.docx]

**Supplementary Materials**

**Fig S1.** Regions of interest from p53-stained endoscopic biopsies classified as “equivocal” by the expert panel, reflecting lack of interobserver agreement. A) Isolated single-crypt involvement (arrow). B. Mosaic p53 expression pattern with abrupt intensity variations.

**Fig S2.** Illustrative examples with morphology resembling NDBE, where subtle focal aberrant p53-IHC patterns (overexpression A, double clone B, null mutation C) prompted re-evaluation by multiple individual pathologists, resulting in diagnostic reclassification.
